# Prevalence of gastrointestinal parasites in bonnet macaque and possible consequences of their unmanaged relocations

**DOI:** 10.1371/journal.pone.0207495

**Published:** 2018-11-15

**Authors:** Shanthala Kumar, Palanisamy Sundararaj, Honnavalli N. Kumara, Arijit Pal, K. Santhosh, S. Vinoth

**Affiliations:** 1 Unit of Nematology-Department of Zoology, Bharathiar University, Coimbatore, Tamil Nadu, India; 2 Department of Conservation Biology, Sálim Ali Centre for Ornithology and Natural History, Coimbatore, Tamil Nadu, India; Centre for Cellular and Molecular Biology, INDIA

## Abstract

Relocation is one of the mitigating measures taken by either local people or related officers to reduce the human-bonnet macaque *Macaca radiata* conflict in India. The review on relocations of primates in India indicates that monkeys are unscreened for diseases or gastrointestinal parasites (henceforth endoparasites) before relocation. We collected 161 spatial samples from 20 groups of bonnet macaque across their distribution range in south India and 205 temporal samples from a group in Chiksuli in the central Western Ghats. The isolation of endoparasite eggs/cysts from the fecal samples was by the centrifugation flotation and sedimentation method. All the sampled groups, except one, had an infection of at least one endoparasite taxa, and a total of 21 endoparasite taxon were recorded. The number of helminth taxon (16) were more than protozoan (5), further, among helminths, nematodes (11) were more common than cestodes (5). Although the prevalence of *Ascaris* sp. (26.0%), *Strongyloides* sp. (13.0%), and *Coccidia* sp. (13.0%) were greater, the load of *Entamoeba coli*, *Giardia* sp., *Dipylidium caninum* and *Diphyllobothrium* sp. were very high. Distant groups had more similarity in composition of endoparasites taxon than closely located groups. Among all the variables, the degree of provisioning was the topmost determinant factor for the endoparasite taxon richness and their load. Temporal sampling indicates that the endoparasite infection remains continuous throughout the year. Monthly rainfall and average maximum temperature in the month did not influence the endoparasite richness. A total of 17 taxon of helminths and four-taxon of protozoan were recorded. The prevalence of *Oesophagostomum* sp., and *Strongyloides* sp., and mean egg load of Spirurids and *Trichuris* sp. was higher than other endoparasite taxon. The overall endoparasite load and helminth load was higher in immatures than adults, where, adult females had the highest protozoan load in the monsoon. The findings indicate that relocation of commensal bonnet macaque to wild habitat can possible to lead transmission of novel endoparasites that can affect their population. Thus, we suggest avoidance of such relocations, however, if inevitable the captured animals need to be screened and treated for diseases and endoparasites before relocations.

## 1. Introduction

Commensalism is an association between two species, where one species is benefited but does not harm or benefit the other [1, 2]. However, the term used in the present context of primates is that they live in association with humans and acquire food and shelter at some cost to humans [3]. Of the 22 known primate species in India [4], bonnet macaque *Macaca radiata*, rhesus macaque *Macaca mulatta*, long-tailed macaque *Macaca fascicularis* and Hanuman langur *Semnopithicus* sp. have adapted to live in a human-dominated landscape, and thus they are the major commensal primates in India [3, 5, 6]. Commensal primates often come into direct contact with humans in their distribution range which leads to human-primate conflict [7]. Many commensal primates are considered as pests [8], since they raid and damage crops, and cause significant economic loss to farmers [9]. Further, they are considered as a menace across their natural range as they snatch food and injure people, raid homes and cause damage to households [10]. As such conflict has been a part of the ecosystem for a long time, people have been taking their own measures to keep the animals away from human habitations or agriculture fields [11–13]. In India, periodic hunting of crop raiding or wild animals otherwise in conflict with humans were in practice during the colonial period [14, 15]. After the Indian Wildlife Protection Act-1972, rules were framed to protect wild animals which included the protection of animals from relocating or killing [16]. Nevertheless, the act permits the killing or capturing of animals that are listed as ‘vermin’ [17].

In the recent past, the intensity of damage caused by monkeys has been perceived to be severe [8, 9, 18]. Consequently, people have been taking their own steps to reduce their losses [10, 19, 20]. Although, people rarely consider drastic retaliatory steps like killing monkeys, the most common technique used is chased them away or relocate the problematic monkeys to distant places [10, 19, 20]. Often, the captured monkeys are released to nearby forest areas including protected areas [6, 10]. Since these biodiversity-rich protected areas are rich with many threatened, endemic and range-restricted species, the effects of such relocated macaques on these native species are not known in India [21, 22]. If the monkeys are infected with parasites including gastrointestinal parasites, such relocations of infected monkeys might transmit those endoparasites to wild inhabitants where those infections can become potentially lethal [23–25]. To evaluate such threats from the relocation of commensal primates, understanding the practice of relocation and prevalence of endoparasites in different habitat condition is crucial.

We examined the relocation practice of primates in India and selected the most commonly relocated primate species, i.e. bonnet macaque for the current study. We made a one-time assessment of endoparasite prevalence in bonnet macaque groups in different habitat conditions, and year-round monitoring of endoparasite prevalence was done in one of the selected groups to understand the seasonality. In the end, we relate the possible relocation of novel endoparasites to the wild by relocating the commensal bonnet macaques.

## 2. Materials and methods

### 2.1. Study site

The bonnet macaque is confined to southern peninsular India and ranges from south of the river Tapti on the west, and the Godavari on the east, including Maharashtra, Goa, Karnataka, Kerala, Tamil Nadu, Telangana and Andhra Pradesh [6, 26, 27]. There are three major landscape units covering the distribution range of bonnet macaque include the Western Ghats, Deccan Plateau, and the Eastern Ghats. Among them, the Western Ghats is one of the biodiversity hotspots including many endemic and endangered species.

### 2.2. Data on the relocation of primates in India

We collated information on the relocation of monkeys from published scientific papers, newspapers, and unpublished reports for the last thirty years (1988–2017) that were available in online sources. We also witnessed relocations of monkeys in different parts of south India during our field studies between 1995 and 2015. The information on relocation included the name of the primate species, year of relocation, details on the location of capture, reason for the relocation, method of capturing, number of individuals captured, the involvement of people and officials in the capturing process, screening of animals for health and endoparasites and details on the relocated locations.

### 2.3. Spatial sampling of bonnet macaque for fecal matter for endoparasites

**Fig 1:**
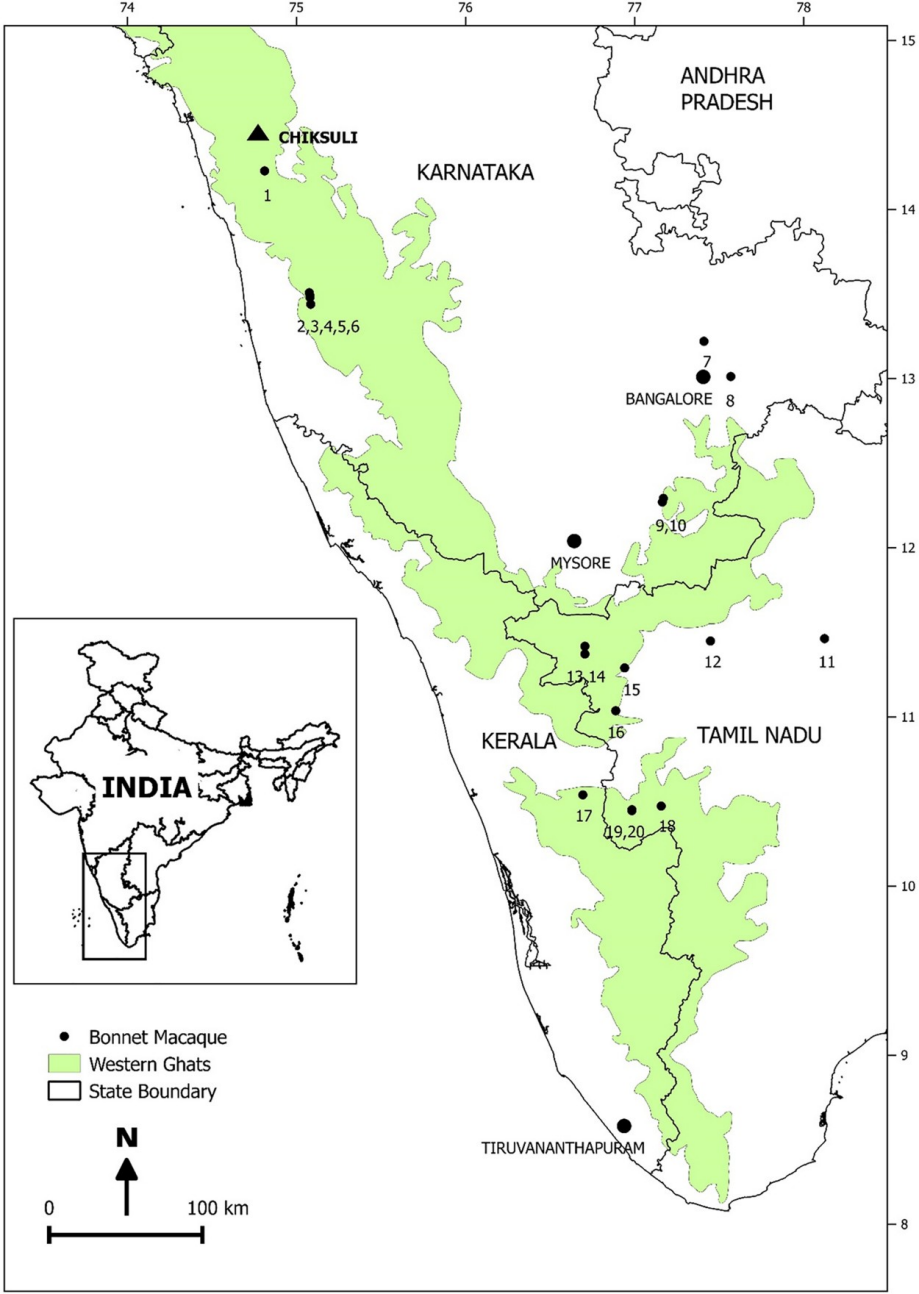
Locations of bonnet macaque groups sampled for fecal samples in south India. Republished from [The India Biodiversity Portal processed using QGIS] under a CC BY license, with permission from [The India Biodiversity Portal and QGIS Team], original copyright [The India Biodiversity Portal: 2004 and QGIS:1991].

We visited different locations of Karnataka, Tamil Nadu and Kerala between November 2014 and May 2015 based on the sites of bonnet macaque reported in Kurup [28] and Kumara et al. [6, 29] (Table 1, Fig 1). During the visit, for every sighting of macaque group, we recorded the group size, location of the group, habitat type, forest type, and degree of provisioning. Since the individual identification was not possible, we planned to follow the group for a day to avoid multiple samples from the same individual. However, despite three to four days of following each of about 70 groups, we could not obtain fecal samples from many groups due to the thick forest canopy, rugged terrain or shyness of the group. We further increased our efforts to improve our sample size for each group, but we were able to obtain fecal samples for only 20 groups that too few samples for some groups. Thus, we treated all collected samples as independent in our analysis. On notice of fresh defecation, 2 g of faeces were weighed, fixed in vials with 10% formalin solution and stored at room temperature. Each vial was labeled with group ID, date and sample number.

**Table 1:** Group size and habitat characteristics of the locations of sampled bonnet macaque groups for the collection of fecal samples.

Location	Location no. in the map	Altitude (m asl)	Group size	Major habitat/ vegetation	Group type	Exposure to humans or degree of provision
AGHP1	2	652	27	EG	Road Side	Very High
AGHP12	3	391	24	EG	Road Side	Low
AGHP4	4	526	20	EG	Road Side	Medium
AKATTI	19	948	52	DDF	Forest	Medium
FPOOTY	13	2300	12	UR	Town	Medium
IISC	7	934	11	UR	Town	High
JOGA	1	579	23	DDF	Tourist/Temp	High
LVOOTY	14	2250	21	Village	Road Side	Low
METTUPALAYAM	15	337	45	UR	Town	Very High
NANDIHILL	8	1478	45	Scrub	Tourist/Temp	High
PACHAMEMT	16	240	58	Village	Road Side	High
PARAMBIKULAM	17	950	23	EG	Forest	Low
S-NADI	5	130	22	EG	Forest	Low
S-NADI CAMP	6	130	23	EG	Forest	High
GAGANACHUKKI	9	736	16	Scrub	Tourist/Temp	Medium
SATTEGALA	10	700	15	Scrub	Road Side	Medium
TRITEMPLE	18	500	34	EG	Tourist/Temp	Medium
VALPARAI	20	650	10	Scrub	Road Side	Medium
VTEMPLE	12	400	55	Village	Tourist/Temp	High
YERCAAD	11	1515	48	DDF	Road Side	High

EG: Evergreen Forest; DDF: Dry Deciduous Forest; UR: Urban. Altitude: The geocoordinates of each group sampled for fecal matter were recorded using handheld global position system GARMIN eTrex. Group size: During sample collection, the number of individuals in the group was counted but due to time constraints group counts were not 100 percent accurate. Each group was counted four to five times by two observers standing on different sides. The maximum count of individuals agreed to by both observers was considered to be the group size. Vegetation: The classification of major vegetation was based on Champion and Seth [30]. The major vegetation or habitat type of each sampling group location was recorded as: evergreen forest, deciduous forest, scrub forest, village and urban. Group (microhabitat) type: The microhabitat of the exact location was further specified as: forest, roadside, tourist spot- temple, and town. The degree of provisioning: We recorded the frequency of all the food resources obtained from different resources by scanning all the individuals for every 30 minutes while collecting the fecal samples. The frequency of all the three days was pooled and calculated the percent frequency of each type of food resources. Using this information we broadly divided the groups into four categories as low, medium, high and very high. If they fed primarily on food resources from the forest or natural trees was considered low. If they fed < 25% on food resources from nonforest areas it was classified as medium. If they fed > 25% and < 75% on human resources (crop, fruits from orchards, human handouts and fallen food on the roadside) it was classified as high. And if they occupied temple or tourist spots and fed on human handouts >75% of the time is was considered very high.

### 2.4. Temporal sampling of bonnet macaque for fecal matter for endoparasites

We selected a forest group of 32 bonnet macaques at Chiksuli in the forests of Sirsi–Honnavara, Karnataka (Fig 1). The group was followed for two months and identified the individuals. We then collected the fecal samples with individual identity from April 2015 to March 2016. We followed the group for three to four days in each month and collected the fecal samples.

### 2.5. Analysis

We synthesized the information on the relocation of primates in India using the pooled data from our observations and available published information.

Laboratory analysis of fecal samples: The specific gravity of endoparasite eggs ranged between 1.08 and 1.27 [31]. The endoparasite eggs and cysts were isolated from the fecal samples, in the laboratory by using flotation concentration and sedimentation techniques [32]. Both techniques were implemented to maximize the detection of all possible endoparasites in the samples. A McMaster’s counting chamber was used to quantify the number of eggs per gram of each endoparasite species in the feces [33].

Flotation concentration method: For each sample, one gram of the fecal sample was taken in a 15 ml Torson centrifuge tube, and 10 ml of distilled water was added to it. Then, the content was homogenized using a glass rod and mixed thoroughly using vortex for 10 min. The mixture was filtered using cheesecloth. The volume of filtrate was increased with distilled water up to 15ml and centrifuged at 1800 rpm for 10 minutes. The supernatant was discarded, and 10 ml saturated sucrose solution (1.3 g/ml) was added to the pellet and thoroughly mixed. The volume of the mixture was increased with a sucrose solution up to 14.5 ml. The mixture was centrifuged at 4000 rpm for about 10 minutes. The upper layer of the mixture was taken and deposited in both the chambers (0.3 ml) of McMaster’s chamber using transfer pipettes and allowed to sit for five minutes in order to let eggs to float to the surface. Finally, eggs were counted a light microscope (Lynx PH-100, LM-52-1804/SL.No. 100044) with a 10X objective.

Sedimentation method: One gram of the fecal sample was taken in a 15 ml Torson centrifuge tube, and 10 ml of distilled water was added to it. The content in the tube was homogenized using a glass rod and mixed thoroughly using vortex for 10min. The mixture was drained using cheesecloth. The filtrate volume was increased to 15ml with distilled water and centrifuged at 1800 rpm for about 10 minutes. Then, the supernatant was discarded, and 10 ml soap solution (specific gravity 0.002) was added to the pellet. The content was centrifuged at 5000 rpm for five minutes. The supernatant was discarded after leaving a few drops of suspension on the sediment pellet. This sediment mixture was taken and deposited in one of the McMaster’s chambers and was observed under the microscope for egg identification and counting.

Each grid of the McMaster’s slide was separately photographed and stored with ID using a microscope camera (ISH500) with the help of IS Capture 3.6.6 software (ISCapture.ink). Identification of eggs, oocysts, and larvae was made based on published identification keys [34–38]. For each analysed sample, the endoparasite name, presence information either from egg or larvae, and count of egg or oocysts were recorded.

Endoparasite richness is a number of endoparasite taxa recorded in the fecal samples of each group of bonnet macaques. The number of endoparasite taxon in each sample in a specific habitat condition was pooled and used to calculate mean endoparasite richness. Endoparasite abundance is defined as the total number of eggs or cysts present in each sample. Endoparasite prevalence is the percent of the samples of the total samples for each group or month having at least one taxon of endoparasite.

**Fig 2:**
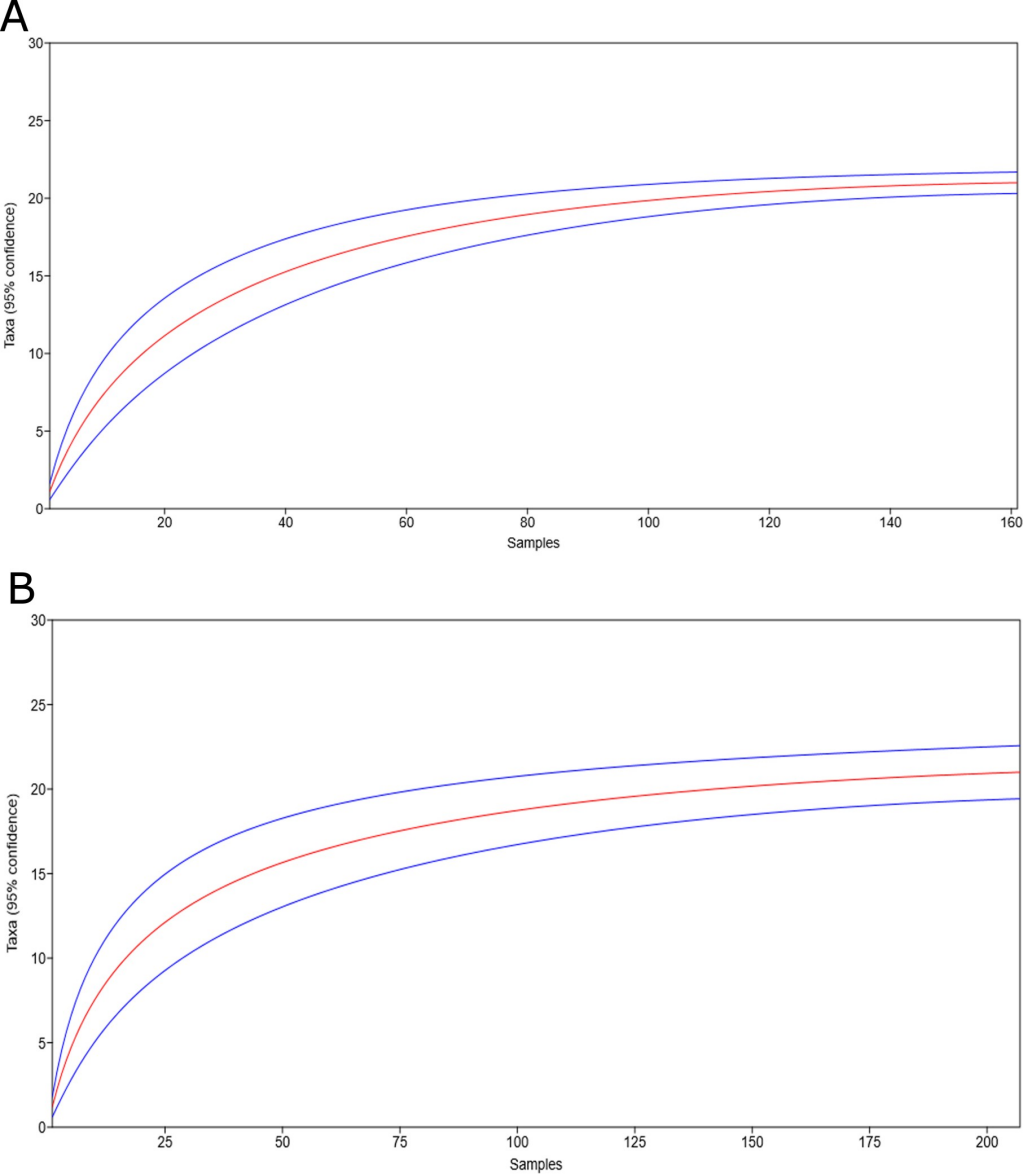
**Rarefaction curve generated for a number of endoparasite taxa against a number of fecal samples of the bonnet macaque** a. Spatial samples from twenty locations, and b. Temporal samples of the group at Chiksuli in the central Western Ghats.

Since the number of fecal samples obtained for some of the sampled groups or monthly smaples of the group at Chiksuli at central Western Ghats was few, we used sample-based rarefaction curves to determine the adequacy of the sampling in detecting the endoparasite species by using the PAST (Fig 2) [39]. The obtained expected taxon richness (*S*_exp_) was used for the further analyses.

Spatial data: The mean abundance of eggs/cysts load between the groups, and the mean number of taxon and the mean abundance of eggs/cysts between vegetation-habitat types, group types and degree of provisioning were tested with ANOVA The Spearman rho correlation was used to check the relationship between group size, altitude and endoparasite richness, and their abundance. The beta diversity of endoparasite taxa in sampled groups were calculated by ‘betapart’ package [40] in the R environment [41]. We constructed the matrix of presence/absence of endoparasite taxa for each group location and constructed the dendrogram for the species similarity in PAST [40].

The endoparasite prevalence, number of endoparasite species, and the total number of eggs, number of protozoan and number of helminths in per gram of fecal sample in each group (N = 20) were considered as five response variables. The predictability of those response variables was tested against five environmental and ecological factors viz. altitude of the group location (AL), group size (GS), group type (locality where a group is situated—GT), vegetation type of the habitat (VG) and degree of provision (PR). Among them, GT, VG, and PR were categorical variables, which are numerically dummy coded according to the increasing association with human beings. The five factors of GT were coded as evergreen forest (1), dry deciduous forest (2), scrubland (3), village (4) and urban area (5). Four VG types were coded as forest (1), roadside (2), town (3) and tourist place/temple (4), where the PR variables were coded from 1 to 4 according to the degree of provisioning. Prior to analysis, the auto-correlation check was done between all five independent variables, which showed a significant positive correlation between GT and VG (S1 Table). Therefore, GT was excluded as the independent variable and finally AL, GS, VG, and PR were used in GLM (Generalized Linear model) analysis. GLM with Poisson distribution models (link = log) were used to examine the relationship between those independent factors and the response variables, except that the prevalence of endoparasites was fitted in a binomial distribution model (link = logit). We selected a set of ten alternatives multiple regression models to explain the variation in frequency of endoparasites between groups. Those ten priori models were ranked in the basis of AIC_c_ (Akaike’s Information Criterion corrected for small sample size bias) and the weight of the model (w_i_) which indicate the probability of the given model within these sets of models. The model with the lowest AIC_c_ value and highest w_i_ was considered as the most parsimonious model or ‘best’ model. However, models within two AIC_c_ values of the parsimonious model were considered as equally parsimonious as the best model [42].

Temporal data on bonnet macaque group at Chiksuli: The mean endoparasite richness and abundance were computed for each month and season (Monsoon: June-September, Winter: October-January, Summer: February-May). The mean endoparasite richness and abundance was related to monthly rainfall and average maximum temperature of the month using Spearman rho correlation tests. The mean abundance of endoparasites was compared between months, seasons and age-sex categories of individuals using ANOVA. We used SPSS ver. 15 [43] for all the correlation tests and comparisons.

### 2.6. Ethical note

We followed all national and international ethical guidelines during this research. Since the bonnet macaque is a commensal species found in wide range of habitat, permits were obtained from concerned Forest Departments wherever it was required. Forest Department permission No. WL 10-46368/2015, dated: 13.01.2016. CWW, Aranya Bhavan, Thiruvananthapuram and No: A5(4). MIS.CR.3/2010-11, dated: 21.07.2016, APCCF, Aranya Bhavan, Bengaluru.

## 3. Results

### 3.1. Relocation of primates in India

In India, a few species of primates are often relocated to reduce human-primate conflict. Of the 25 instances of relocations between 1988–2017 (S 1), except for one instance of hoolock gibbon (*Hoolock leuconedys)* being relocated from a forest fragment to another habitat, all relocations were of bonnet macaques (N = 13) and rhesus macaques (N = 11). The macaque groups were relocated from various type of habitats, viz. tourist locations including temples (N = 12, 50.0%), urban groups (N = 7, 29.2%) and agriculture fields (N = 3, 12.5%). About 100,000 rhesus macaques were captured and sterilized and relocated to different locations in Himachal Pradesh [44, 45]. On the other hand, rhesus macaques were re-translocated from Sariska Tiger Reserve to avoid endoparasite infection to other native animals. In another instance, bonnet macaques were left in a tied gunny bag near the forest fringes of MM. Hills in Karnataka, they later died [S2 Table]. The monkeys were relocated to nearby forests (N = 18, 72.0%) or roadsides (N = 6, 24.0%), especially along the hilly roads. However, bonnet macaques captured from tourist locations or temples were often released to evergreen forests or shola forests (N = 4 instance) at high altitude. Monkeys were considered as nuisances or pests by local people raising crops or tourists due to raiding, stealing or snatching of food, and aggressive physical interactions like scratching and biting people. In response to complaints from inhabitants, relocation of bonnet macaque from the conflict interfaces was undertaken taken by the Forest Department. However, about 40.0% of capturing and relocation of monkeys was done by local people or by ‘monkey catchers' without forest department guidance or involvement. In none of the translocations was the prescribed protocol for monkey translocation followed. Neither the entire group (with the proper age-sex distribution of individuals) was captured and relocated, nor was screening was done for diseases and endoparasite infections. The monkeys captured were released with skewed sex ratios, and in some cases, unrelated individuals from different groups were released together. Further, the relocated groups were never monitored for any aspects of their health, ecology, behavior, or impact of their release on local fauna, ecosystem, and health of local people or agriculture. In a few cases, monkeys were also relocated to non-suitable habitats like high altitude rainforests having many endemic and endangered species.

### 3.2. Status of endoparasites in spatial samples of bonnet macaque

We collected 161 fecal samples from 20 groups of bonnet macaques, where, the number of samples varied from 2 to 18 per group (S3 Table). The endoparasites were recorded in all the sampled groups except Valparai. However, their prevalence across groups ranged from 33.0% to 100.0% (Table 2). Of the total samples, 66.5% (N = 107) were infected with at least one endoparasite taxa. Of the infected samples, 65.4% (N = 70) had one endoparasite taxa where 34.6% (N = 37) had multiple endoparasite taxa.

**Table 2:** Number of samples and percent prevalence of endoparasites in bonnet macaque in spatial sampling.

Location	No. of samples	Samples with endoparasites	Prevalence (%)	No. of endoparasite taxa (*S*_obs_)	Estimated endoparasite taxa (*S*_est_)	Mean no. of eggs-cysts ±SD	Mean No. of helminth eggs ±SD	Mean no. of protozoan cysts ±SD
AGHP1	3	1	33.3	3	2.9	665.0	45.0	620.0
AGHP12	8	6	75.0	3	6.4	13.5±9.7	19.0±1.4	10.8±11.3
AGHP4	8	3	37.5	3	6.4	5.3±5.1	2.0±1.7	10.0
AKATTI	8	4	50.0	2	6.4	81.3±46.5	0	81.3±46.5
FPOOTY	12	6	50.0	1	8.3	5.2±4.2	5.2±4.2	0
IISC	6	4	66.7	3	5.2	215.3±123.9	4.5±0.7	213.0±121.3
JOGA	12	11	91.7	8	8.3	30.0±35.9	26.4±36.9	20.0±0.0
LVOOTY	13	8	61.5	8	8.7	81.5±104.7	36.0±36.2	133.3±161.7
METTUPALAYAM	6	4	66.7	7	5.2	489.8±172.1	55.5±37.7	434.3±134.5
NANDIHILL	10	8	80.0	5	7.4	33.0±19.8	27.9±17.8	20.5±0.7
PACHAMEMT	6	6	100.0	10	5.2	134.5±156.6	105.7±127.4	43.3±25.5
PARAMBIKULAM	2	2	100.0	1	2.1	25.5±3.5	25.5±3.5	0
S-NADI	14	8	57.1	2	9.1	20.9±30.5	4.6±4.6	65.0±7.1
S-NADI CAMP	18	16	88.9	4	10.5	38.8±23.7	38.8±23.7	0
GAGANACHUKKI	4	4	100.0	4	3.8	35.0±38.2	35.0±38.1	0
SATTEGALA	5	5	100.0	6	4.5	48.4±53.7	67.0±63.9	20.5±27.6
TRITEMPLE	4	2	50.0	2	3.8	25.0±1.4	25.0±1.4	0
VALPARAI	2	0	00	0	2.1	0	0	0
VTEMPLE	12	4	33.3	2	8.3	94.5±82.1	3.0±2.8	93.0±83.8
YERCAAD	8	5	62.5	6	6.4	112.6±167.3	123.3±166.7	23.3±15.3
	161	107	66.5			76.7±131.5	36.8±57.9	121.4±163.9

### 3.3. Endoparasite species composition in spatial samples of bonnet macaque

**Fig 3:**
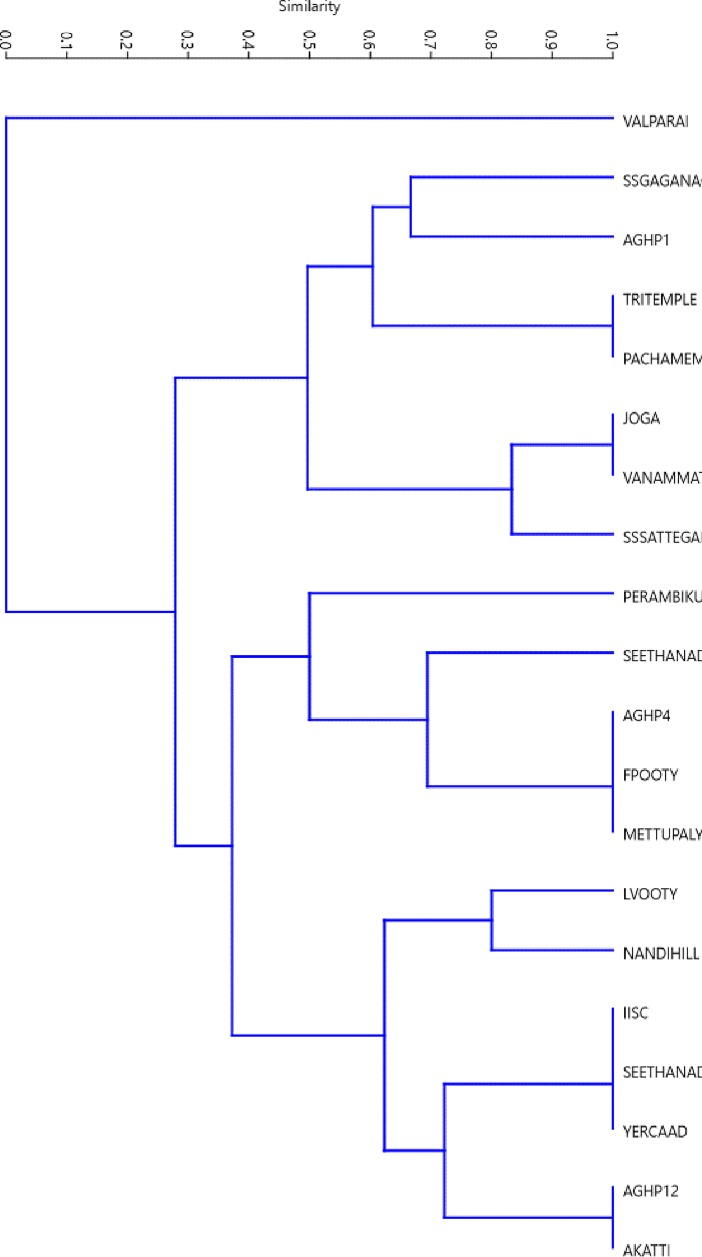
Simpson similarity index showing clustering of geographical locations of bonnet macaque groups based on the composition of endoparasite taxon.

Of the 21 taxa of endoparasites recorded, 16 were helminths that include 12 nematodes and five cestodes, and five protozoans. However, the number of endoparasite taxa varied from 1 to 10 between the groups (Tables 2 and 3). The most common endoparasites were two nematodes, i.e., *Ascaris* sp. (26.0%) and *Strongyloides* sp. (13.0%), and one protozoan (*Coccidia* sp.: 13.0%) (Table 3). Among endoparasite taxa, the species richness of nematodes was highest, though the mean cysts of protozoans and eggs of cestodes were higher in the infected samples. Among nematodes, the mean number of eggs of *Trichuris* sp. (42.8±40.5_SD_) was highest in the infected samples, where among cestodes, *Dipylidium caninum* (140.0) and *Diphyllobothrium* sp. (114.5±163.9_SD),_ and among protozoans, *Entamoeba coli* (350.2±301.4_SD_) and *Giardia* sp. (235.5±6.4_SD_) had the highest number of eggs/cysts. The beta diversity of endoparasite species in each group showed Simpson pair-wise dissimilarity for replacement, Sorenson pair-wise dissimilarity for a nested fraction and Sorenson pair-wise dissimilarity for overall (0.819, 0.858 and 0.905 respectively). There was no relation between the geographical distances and the composition of endoparasite taxa for sampled groups (*r* = -0.015, p = 0.558), perhaps the groups in distant places had more similarity in composition of endoparasite taxa than did closely located groups (Fig 3).

**Table 3:** Endoparasite taxon and their prevalence in spatial samples of bonnet macaque (N = 161).

No.	Endoparasites	Detected in no. of groups	%groups infected	No. of positive samples	Prevalence (%)	Mean eggs/cysts in infected samples
	**Nematodes**					
1	Spirurids	1	5	2	1.2	35.0±1.4
2	*Strongyloides* sp.	9	45	21	13.0	23.1±26.7
3	*Trichuris* sp.	1	5	6	3.7	42.8±40.5
4	*Ancylostoma* sp.	1	5	2	1.2	31.0±1.4
5	*Bunostomum* sp.	3	15	7	4.4	40.1±45.8
6	*Haemonchus* sp.	2	10	2	1.2	20.0
7	*Ascaris* sp.	11	55	41	25.5	11.8±20.1
8	*Toxocara* sp.	3	15	3	1.9	8.7±10.0
9	*Enterobius vermicularis*	3	15	6	3.7	10.7±11.7
10	*Trichostrongylus* sp.	6	30	14	8.7	9.1±8.4
11	*Oesophagostomum* sp.	3	15	5	3.1	23.8±28.3
	**Cestodes**					
1	*Diphyllobothrium* sp.	3	15	4	2.5	114.5±163.9
2	*Moniezia* sp.	5	25	9	5.6	26.2±10.8
3	*Hymenolepis nana*	2	10	3	1.9	42.3±67.3
4	*Taenia* sp.	3	15	8	5.0	40.0±19.3
5	*Dipylidium caninum*	1	5	1	0.6	140.0
	**Protozoa**					
1	*Coccidia* sp.	8	40	21	13.0	67.7±74.6
2	*Balantidium coli*	8	40	14	8.7	59.4±37.9
3	*Entamoeba coli*	4	20	5	3.1	350.2±307.4
4	*Entamoeba histolytica*	2	10	3	1.9	126.7±167.7
5	*Giardia* sp.	1	5	2	1.2	235.5±6.4

### 3.4. Endoparasite abundance in spatial samples of bonnet macaque

The abundance of egg/cysts load varied significantly between the groups (range: 5.2±4.2_SD_ eggs-cysts/g in FPOoty and 665.0 eggs-cysts/g in AGHP1: F_17, 89_ = 13.149, p < 0.001). Similarly, the protozoan cysts (range: 10.0 cysts/g in AGHP4 and 620.0 cysts/g in AGHP1: F_13, 26_ = 9.528, p < 0.001) was also varied significantly between the groups (Table 2), however, helminth eggs (range: 2.0±1.7 eggs/g in AGHP4 and 123.3±166.7_SD_ eggs/g in PachameMT: F_16, 74_ = 1.719, p = 0.06) did not vary.

The overall load increased with increase in group size (*r*_s_ = 0.466, df = 20, p < .05), otherwise group size did not influence the number of endoparasite taxa, prevalence, protozoan cysts, and helminth eggs across the groups (Table 4). Further altitude did not influence any of the endoparasite parameters across the groups.

**Table 4:** The Spearman’s rho correlation tests between altitude, group size and number of endoparasite taxon as independent parameter and number of endoparasite taxon in the population, mean number of eggs/cysts, protozoan cysts and helminth eggs as a dependent parameter.

Parameter	Altitude	Group size
No. of endoparasite taxon	*r*_s_ = -0.114, df = 20, p = 0.631	*r*_s_ = 0.116 df = 20, p = 0.626
Endoparasite prevalence	*r*_s_ = -0.023, df = 20, p = 0.924	*r*_s_ = 0.019, df = 20, p = 0.936
Mean number of eggs/cysts	*r*_s_ = 0.006, df = 20, p = 0.980	*r*_s_ = 0.466, df = 20, p <0.03
Number of protozoan cysts	*r*_s_ = -0.029, df = 20, p = 0.902	*r*_s_ = 0.363, df = 20, p = 0.116
Number of helminth eggs	*r*_s_ = 0.000, df = 20, p = 1.000	*r*_s_ = 0.288, df = 20, p = 0.218

The mean number of endoparasite taxon significantly varied between the vegetation types (F_4,102_ = 5.781, p < .001) (Table 5). The urban monkeys had significantly higher overall egg/cysts load (F_4,102_ = 5.196, p < .001), and protozoan cysts (F_4,35_ = 6.170, p < .001) than in other vegetation types. Whereas helminth load did not differ between the vegetation types (F_4,86_ = 1.404, p = 0.239) but differed between group types (F_3,87_ = 2.919, p <0.05) whereas roadside groups had a higher load than in the other groups. The helminth load was more than double in groups exposed to a higher degree of provisioning than the lower degree of provisioning, but the difference is not significant (F_3,87_ = 1.455, p = 0.233). The town groups had significantly higher endoparasite taxon (F_3,103_ = 4.024, p < .01), overall load (F_3,103_ = 7.069, p < .001) and protozoan load (F_3,36_ = 7.753, p < .001) than other group types. Similarly, groups exposed to a very high degree of provisioning had a significantly higher number of endoparasite taxon (F_3,103_ = 5.619, p < .01), overall load (F_3,103_ = 49.708, p < .001) and protozoan load (F_3,36_ = 24.658, p < .001) than the groups with less provisioning.

**Table 5:** Mean a number of eggs/cysts of endoparasites in bonnet macaque in different habitat conditions.

Parameters	Mean no. of taxon ±SD(N)	Mean no. of eggs-cysts ±SD(N)	Mean no. of helminth eggs ±SD (N)	Mean no. of protozoa cysts ±SD (N)
**Vegetation-Habitat**				
Evergreen Forest	1.1±0.4 (36)	44.4±109.1 (36)	24.9±23.4 (32)	100.4±211.5 (8)
Deciduous Forest	1.4±0.6 (20)	60.9±90.9 (20)	52.2±94.3 (15)	48.3±43.0 (9)
Scrub Forest	1.8±1.0 (19)	36.6±33.1 (19)	36.1±33.9 (17)	20.5±15.9 (4)
Village	2.6±2.2 (14)	102.1±116.2 (18)	59.5±89.4 (15)	85.9±94.6 (11)
Urban	2.1±1.3 (14)	203.6±231.9 (14)	21.8±31.8 (12)	323.6±167.4 (8)
	F_4,102_ = 5.781, p < .001	F_4,102_ = 5.196, p < .001	F_4,86_ = 1.404, p = 0.239	F_4,35_ = 6.170, p < .001
**Group type**				
Forest	1.1±0.3 (30)	38.8±32.9 (30)	27.2±24.3 (26)	75.8±37.1 (6)
Road Side	2.0±1.8 (34)	89.0±149.1 (34)	64.2±95.2 (26)	75.4±153.7 (18)
Tourist/Temple	1.7±0.9 (29)	40.1±43.9 (29)	26.3±28.8 (27)	56.6±67.3 (8)
Town	2.1±1.3 (14)	203.6±231.9 (14)	21.8±31.8 (12)	323.6±167.4 (8)
	F_3,103_ = 4.024, p < .01	F_3,103_ = 7.069, p < .001	F_3,87_ = 2.919, p <0.05	F_3,36_ = 7.753, p < .001
**Degree of provision**				
Low	1.3±0.6 (24)	39.6±67.5 (24)	19.9±25.6 (19)	63.7±99.0 (9)
Medium	1.5±0.8 (24)	33.5±41.7 (24)	23.8±36.2 (18)	53.7±48.9 (7)
High	1.8±1.5 (54)	70.8±96.1 (54)	46.4±71.9 (49)	81.5±96.7 (19)
Very High	3.6±0.5 (5)	524.8±168.4 (5)	53.4±33.0 (5)	471.4±143.0 (5)
	F_3,103_ = 5.619, p < .01	F_3,103_ = 49.708, p < .001	F_3,87_ = 1.455, p = 0.233	F_3,36_ = 24.658, p < .001

### 3.5. Determinants of endoparasite richness, prevalence, and abundance in spatial samples of bonnet macaque

A parsimonious model (PR+VG) with two determining variables *viz*. provision and vegetation were considered as mediating factors for both distribution of endoparasite egg/cysts and protozoan in the fecal samples (Table 6). In case of distribution of egg/cysts of endoparasite, ‘PR+VG’ model (R^2^ = 0.67, w_i_ = 1, p<0.001) predicted the high influence of the degree of provisioning (*β* = 0.95 ± 0.04_SE_) followed by vegetation (*β* = -0.16± 0.02_SE_). Similarly, it also shows that provisioning (*β* = 1.52 ± 0.05_SE_) and vegetation (*β* = 0.23 ± 0.02_SE_) determined the distribution of protozoan in the bonnet groups. ‘GS + PR’ (R^2^ = 0.14, w_i_ = 0.96, p<0.001) was the most parsimonious model which showed that provisioning (*β* = 0.18 ± 0.06_SE_) followed by group size (*β* = 0.02 ± 0.01_SE_) had the highest effect on the distribution of helminths. However, in case of the distribution of the number of endoparasite species, four models with all four independents were ranked as parsimonious models (Δ AIC_c_<2) that include altitude, group size, vegetation and provision as predicting variables, where the influence of provisioning (R^2^ = 0.01, w_i_ = 0.18, p<0.001, *β* = -0.40± 0.37_SE_) played the highest role in the prevalence of endoparasites in macaque groups (Table 7). For prevalence of endoparasite species, again four independent variables ranked as parsimonious models with lowest AIC_c_. Among them, the best fit model (R^2^ = 0.02, w_i_ = 0.23, p = 0.19) showed group size (*β* = 0.02± 0.03_SE_) as the most determinant factor for the prevalence of endoparasite species. In overall, provisioning was the common predicting variable for the distribution of endoparasites in sampled bonnet groups. However, along with the very low R^2^_McFadden_ value (<0.5), no parsimonious model for the number of species and prevalence of endoparasites significantly differed from the null model, which made these models unlikely.

**Table 6:** Summary of the model selection procedure for covariates influencing the distribution of egg, protozoan, and helminths in bonnet macaques, with R^2^_McFadden_ and corresponding p-value, β coefficients and associated standard errors.

Covariates	K	R^2^	p	w_i_	AIC_c_	Δ AIC_c_	β coefficient	SE
**Overall load distribution models**								
**PR+VG**	3	0.67	<0.001	1	522.19	**0.00**	**0.95, 0.16**	0.04, 0.02
PR	2	0.62	<0.001	0.00	605.26	83.08	**1.00**	0.04
GS+PR	3	0.62	<0.001	0.00	607.75	85.56	<0.01, 1.00	<0.01, 0.04
AT+VG	3	0.31	<0.001	0.00	1094.27	572.09	<-0.001, 0.38	<0.001, 0.20
VG	2	0.18	<0.001	0.00	1286.58	764.39	0.32	0.02
AT+GS	3	0.12	<0.001	0.00	1390.63	868.44	<-0.001, <0.01	<0.001, <0.01
GS	2	0.09	<0.001	0.00	1432.96	910.77	0.02	<0.01
AT	2	0.04	<0.001	0.00	1503.45	981.26	<-0.001	<-0.001
**Protozoan load distribution models**								
**PR+VG**	3	0.67	<0.001	1	677.63	**0.00**	PR:1.52, VG:0.23	0.05, 0.02
GS+PR	3	0.63	<0.001	0.00	773.07	95.44	GS: <-0.01, PR:1.63	<0.001, 0.05
PR	2	0.62	<0.001	0.00	780.55	102.92	PR:1.59	0.52
AT+VG	3	0.27	<0.001	0.00	1500.49	822.85	AT: <-0.001, VG:0.52	<0.001, 0.02
VG	2	0.17	<0.001	0.00	1697.27	1019.63	VG:0.45	0.03
AT+GS	3	0.06	<0.001	0.00	1916.26	1238.63	AT: <-0.001, GS:0.02	<0.001, <0.001
GS	2	0.04	<0.001	0.00	1968.96	1291.33	GS:0.02	0.03
AT	2	0.04	<0.001	0.00	1971.19	1293.55	AT: <-0.001	<0.001, <0.01
**Helminth load distribution models**								
**GS+PR**	3	0.14	<0.001	0.96	480.19	**0.00**	GS:0.02, PR:0.18	<0.01, 0.06
GS	2	0.12	<0.001	0.03	487.39	7.20	GS:0.02	<0.01
AT+GS	3	0.13	<0.001	0.02	488.16	7.96	AT:<-0.001, GS:0.02	<0.001, <0.001
PR+VG	3	0.08	<0.001	0.00	513.18	32.98	PR:0.28, VG:0.07	0.05, 0.03
PR	2	0.07	<0.001	0.00	515.19	34.99	PR:0.03	<0.001
AT+VG	3	0.06	<0.001	0.00	524.48	44.27	AT:<-0.001, VG:0.16	<0.001, <0.001
VG	2	0.03	<0.001	0.00	539.39	59.19	VG:0.12	<0.001
AT	2	0.01	<0.001	0.00	547.48	67.28	AT:<-0.001	0.01

AL: altitude; GS: group size; PR: degree of provisioning, VG: vegetation; K: number of parameters estimated by the model; R^2^: McFadden coefficient of determination; w_i_: model weight; AIC_c_: AIC corrected for small sample size biased, and Δ AIC_c_: difference of AIC_c_ value from the lowest AIC_c_, where bold values represent the parsimonious model (Δ AIC_c_<2).

**Table 7:** Summary of the model selection procedure for covariates influencing the distribution of endoparasite taxon and their prevalence in bonnet macaques, with R^2^_McFadden_ and corresponding p-value, β coefficients and associated standard errors.

Covariates	K	R^2^	p	w_i_	AIC_c_	Δ AIC_c_	β coefficient	SE
**Endoparasite taxon distribution models**								
**AT**	2	0.01	<0.001	0.22	78.02	**0.00**	AT: <0.001	<0.001
**GS**	2	0.01	<0.001	0.21	78.12	**0.10**	GS:0.01	0.02
**VG**	2	<0.01	<0.001	0.19	78.34	**0.32**	VG:0.08	0.23
**PR**	2	0.01	<0.001	0.18	78.45	**0.42**	PR:-0.40	0.37
AT+GS	3	0.01	<0.01	0.06	80.70	2.68	AT: <0.001, GS: 0.02	<0.001, 0.02
GS+PR	3	<0.01	<0.01	0.05	81.14	3.11	GS:0.02, PR: -0.15	0.03, 0.41
AT+VG	3	<0.01	<0.001	0.05	81.18	3.16	AT: <0.001, VG:0.02	<0.001, 0.25
PR+VG	3	<0.01	<0.01	0.04	81.46	3.43	PR:-0.08, VG:0.09	0.40, 0.25
**Endoparasite prevalence models**								
**GS**	2	0.02	0.19	0.23	28.58	**0.00**	GS:-0.02	0.03
**PR**	2	<0.01	0.19	0.19	28.93	**0.35**	PR:-0.23	0.55
**AT**	2	<0.01	0.31	0.18	29.05	**0.47**	AT:<-0.001	<0.001
**VG**	2	<0.01	0.41	0.18	29.07	**0.49**	VG:0.03	0.34
AT+GS	3	0.03	0.21	0.06	31.17	2.59	AT:<-0.001, GS: -0.02	<0.001, 0.03
GS+PR	3	0.03	0.27	0.06	31.34	2.76	GS:-0.01, PR: -0.11	0.04, 0.60
PR+VG	3	0.02	0.38	0.05	31.46	2.88	PR:-0.26, VG:0.08	<0.001, 0.38
AT+VG	3	0.02	0.38	0.05	31.56	2.99	AT:<-0.001, VG:0.08	0.57, 0.36

AL: altitude; GS: group size; PR: degree of provisioning, VG: vegetation; K: number of parameters estimated by the model; R^2^: McFadden coefficient of determination; w_i_: model weight; AIC_c_: AIC corrected for small sample size biased, and Δ AIC_c_: difference of AIC_c_ value from the lowest AIC_c_, where bold values represent the parsimonious model (Δ AIC_c_<2).

### 3.6. Status of endoparasites in a temporal sample of bonnet macaque group

We collected 205 fecal samples from the focal study group of bonnet macaques across different months at Chiksuli. The number of fecal samples varied from 5 in November to 42 in April. Of these 140 (68.3%) samples had endoparasite infections (Table 8, S4 Table). A total of 21 endoparasite taxon were recorded that include 17 helminth taxa (14 nematodes and three cestodes), and four protozoan taxa (Table 9). Among them, the prevalence of *Oesophagostomum* sp. (26.8%), *Strongyloides* sp. (19.5%), and *Ascaris* sp. (13.2%) of helminths, and *Coccidia* sp. (9.3%) of protozoa were predominant. The endoparasite prevalence varied from 42.9% in March to 85.0% in July (Table 8). However, the endoparasite richness was highest in April (14 taxa) and May (11 taxa). Monthly rainfall (*r*_s_ = 0.146, df = 12, p = 0.651) and average maximum temperature (*r*_s_ = 0.041, df = 12, p = 0.898) did not influence endoparasite richness.

**Table 8:** Number of samples and percent prevalence of endoparasites in bonnet macaque in Chiksuli.

Month	Average rainfall (mm)	Average high temperature (°C)	No. Samples	Samples with endoparasites	% Prevalence	No. of observed taxon (*S*_obs_)	Estimated endoparasite taxon (*S*_exp_)
June	684.4	26.0	9	6	66.7	7	7.48
July	3008.8	24.1	20	17	85.0	8	11.29
August	1009.7	24.4	18	13	72.2	7	10.67
September	810.3	25.5	15	11	73.3	8	9.69
October	320.2	28.3	11	8	72.7	10	8.27
November	166.1	28.5	5	4	80.0	4	5.61
December	11.2	29.5	28	13	46.4	9	13.00
January	31.2	30.2	6	4	66.7	4	6.14
February	0	31.0	18	13	72.2	8	10.67
March	0	32.0	7	3	42.9	3	6.62
April	29.4	32.0	42	30	71.4	14	13.00
May	59.3	31.0	26	18	69.2	11	13.00
**Total**			**205**	**140**	**68.3**	**21**	

**Table 9:** Endoparasite taxon and their prevalence in temporal samples of bonnet macaque (N = 205).

Sl. No.	Endoparasite taxon	Number of positive samples	Prevalence (%)	Mean eggs/cysts in infected samples
	**Nematodes**			
1	Spirurids	7	3.4	271.4±501.0
2	*Strongylus* sp.	7	3.4	21.4±21.9
3	*Strongyloides* sp.	40	19.5	71.3±140.4
4	*Trichuris sp*.	14	6.8	171.6±296.3
5	*Ancylostoma* sp.	14	6.8	9.9±15.6
6	*Bunostomum* sp.	3	1.5	29.0±14.7
7	*Haemonchus* sp.	3	1.5	19.5±20.3
8	*Ascaris* sp.	27	13.2	23.4±33.2
9	*Oesophagostomum* sp.	55	26.8	23.7±43.6
10	*Toxocara* sp.	4	2.0	31.3±60.5
11	*Enterobius vermicularis*	2	1.0	10.5±13.4
12	*Trichostrongylus* sp.	5	2.4	32.2±13.0
13	*Metastrongylus* sp.	14	6.8	37.9±29.0
14	*Nematodirus* sp.	2	1.0	1.5±0.7
	**Cestodes**			
1	*Moniezia* sp.	1	0.5	20.0
2	*Hymenolepis nana*	5	2.4	47.8±35.7
3	*Taenia* sp.	1	0.5	40.0
	**Protozoa**			
1	*Coccidia* sp.	19	9.3	35.2±42.2
2	*Balantidium coli*	11	5.4	29.8±31.3
3	*Entamoeba coli*	12	5.9	32.1±40.3
4	*Giardia* sp.	2	1.0	29.5±13.4

### 3.7. Endoparasite abundance in a temporal samples of bonnet macaque group

**Fig 4:**
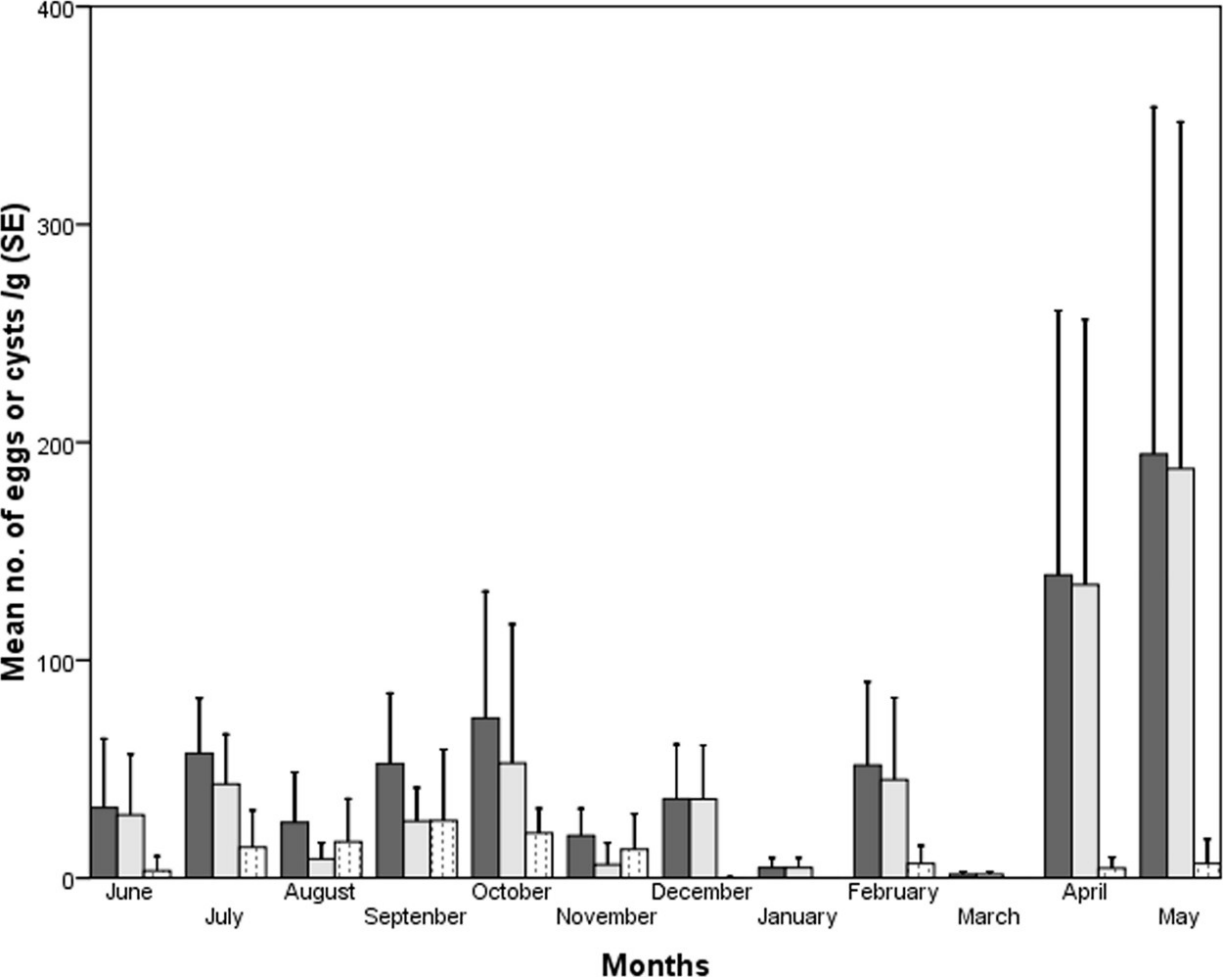
Mean egg/cysts load in fecal samples of bonnet macaque across different months (dark gray: Overall load; light gray: Helminth load; dotted: Protozoan load).

Although the overall egg/cysts and helminth egg load was higher in April and May, and protozoan cysts load in September and October (Fig 4) but did not vary significantly between the months (overall egg/cyst load: F_11,131_ = 1.002, p = 0.448; helminth egg load: F_11,131_ = 1.086, p = 0.377; and protozoan cyst load: F_11,131_ = 1.148, p = 0.330). The average monthly rainfall did not influence the overall load (*r*_s_ = 0.189, df = 12, p = 0.556) and helminth egg load (*r*_s_ = 0.00, df = 12, p = 1.00), but protozoan cysts load (*r*_s_ = 0.707, df = 12, p < 0.01) increased with increase in the rainfall. Similarly, the average maximum temperature of a month did not influence the overall load (*r*_s_ = -0.011, df = 12, p = 0.974) and helminth egg load (*r*_s_ = 0.130, df = 12, p = 0.688), but negatively influenced the protozoan cysts load (*r*_s_ = -0.645, df = 12, p < 0.05).

### 3.8. Endoparasite abundance in age-sex individuals in different seasons temporal samples of bonnet macaque group

**Fig 5:**
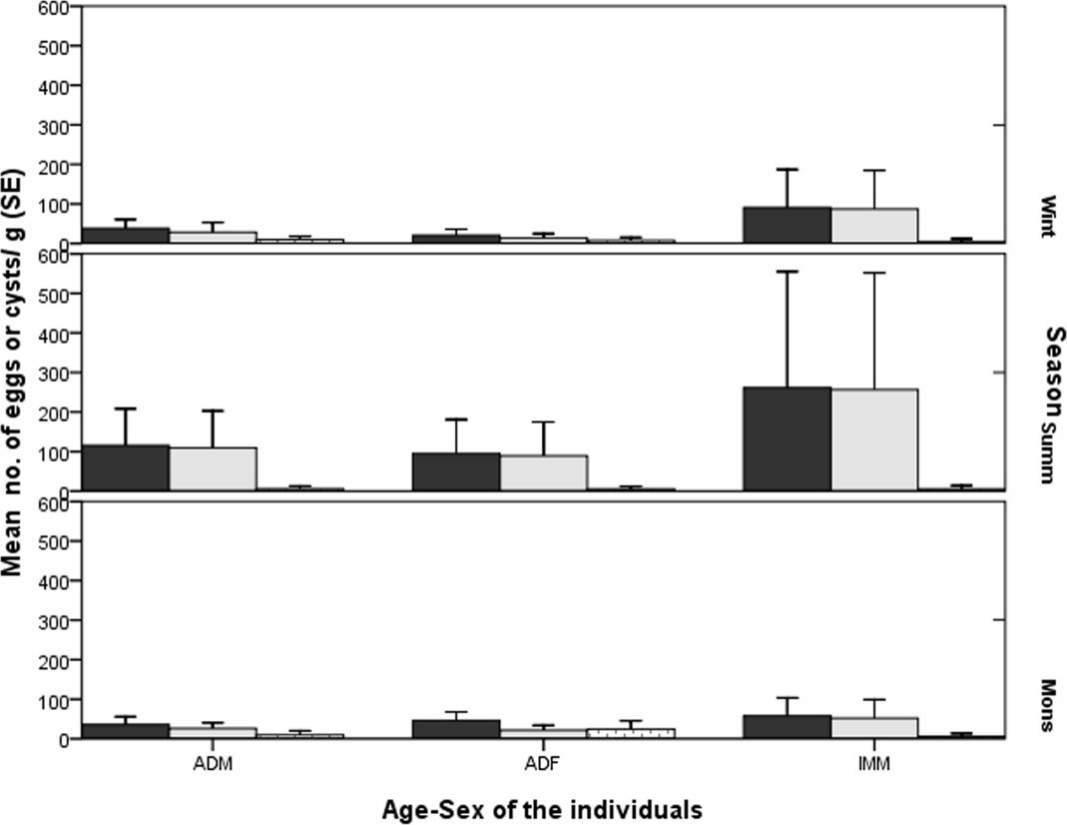
Mean egg/cysts load in fecal samples of different age-sex individuals of bonnet macaques in different seasons (dark gray: Overall load; light gray: Helminth load; dotted: Protozoan load).

The seasonally pooled fecal samples of different age-sex individuals showed immature individuals had higher overall load and helminth load than did adults in all the seasons that too more in the summer (Fig 5). While adult females had higher protozoan cyst load than adult males and immatures in monsoon than in other seasons, the variation in overall load (F_2,140_ = 2.161, p = 0.119), helminth load (F_2,140_ = 2.416, p = 0.09) and protozoan load (F_2,140_ = 0.746, p = 0.476) did not vary significantly between the seasons.

## 4. Discussion

Although relocation of commensal bonnet macaques is commonly used to reduce monkey-human conflict, relocations are often done with screening the animals for diseases or parasites. All of the groups that we sampled were infected by at least one endoparasite taxon, and temporal sampling indicated the persistence of endoparasites in every month. The 21 endoparasite taxon recorded included 16 taxa of helminths and five taxa of protozoans. Among helminths, nematodes (11) were more common than cestodes (5) in spatial sampling. Although the prevalence of *Ascaris* sp., *Strongyloides* sp. and *Coccidia* sp. were highest, the load of *Entamoeba coli*, *Giardia* sp., *Dipylidium caninum* and *Diphyllobothrium* sp. Were also very high. The degree of the provisioning was the topmost determinant for the richness of endoparasite taxa and their load. Temporal sampling revealed that the endoparasite prevalence varied from *ca*. 48.0% to 85.0%, and that species richness was greater in the summer. *Oesophagostomum* sp., *Strongyloides* sp., and *Ascaris* sp. were predominant. The overall endoparasite load and helminth load was more in immature than in adults in all the seasons, and adult females had the highest protozoan load than adult males and immature in the monsoon.

All free-living animals, including primates, act as primary or secondary hosts to endoparasites. Endoparasites and their host species have co-evolved over time for survival in each habitat condition [46–48]. Although, the percent prevalence of endoparasites varied across groups, and seasonally, the total spatial and temporal samples indicate at least *ca*. 68.0% of the macaque population was always infected. This indicates the persistence of endoparasites throughout the year in all the populations was similar to many other primate species, e.g., *Mandrillus sphinx* [49] and *Papio ursinus* [50].

Globally very few species have been screened for endoparasites across their spatial range of distribution. For example, the recording of 61 helminths taxa in opossum *Didelphis virginiana* [51] and 72 helminths taxa in Nearctic and Palearctic populations of *Canis lupus* [52] over their geographical range indicates the importance of spatial sampling in revealing the probable diversity of endoparasites in a species. Although our sampling was only in half of the distribution range of bonnet macaques, the recording of 24 taxa of endoparasites, which include 19 helminths taxon and five protozoan taxa is the first ever report for the species. Except for a few groups like LVOoty, all groups were exposed to a high degree of provisioning in habitats dominated by humans and domestic animals, and these groups had the infection of multiple endoparasite taxa (>3 taxa), including an abundance of cestodes. All the endoparasites recorded in bonnet macaques are also recorded in at least one other primate species in the world (S3 Table). Some of the endoparasite taxa are often reported in many primate species around the globe, e.g., *Strongyloides* sp., *Trichuris* sp., *Ascaris* sp., *Oesophagostomum* sp., *Strogylus* sp., *Balantidium coli*, *Entamoeba coli*, *Entamoeba hystolitica* and *Giardia* sp. Whereas some taxa are rarely recorded in primates, e.g., Spirurids, *Bunostomum* sp., *Haemonchus* sp., *Toxocara* sp., *Metastrogylus* sp., *Nematodirus* sp., *Diphyllobothrium* sp., *Hymenolepis nana*, *Taenia* sp., *Diphylidium caninum*, and *Coccidia* sp. *Bunostomum* sp., *Haemonchus* sp., *Diphylobothrium* sp., *Hymenolepis nana*, *Moniezia* sp., and *Coccidia* sp., are known in *M*. *silenus* [53] and *S*. *johnii* [54] from south India where they are sympatric with the bonnet macaque. Having a direct life cycle, many of these nematodes can transmit the infection from monkeys to man vice versa [55]. Among protozoans *Entamoeba* sp. and *Balatidiunm coli* are pathogenic, large ciliates which infect animals as well as humans [56]. The *Entamoeba* sp. infects directly through water or food and, in heavy infestations can lead to the death of host animal [57].

Anthropocentric activities, like disturbance of the habitat or introduction of highly infected animals with different endoparasites, are the major driving force in spreading of the alien endoparasites. These practices affect the individual’s ability to cope with the multiple infections. For example, primates exposed to disturbed forests due to selective logging, fragmentation and clear felling are shown to have more endoparasites, e.g. *M*. *silenus* [53], *Procolobus rufomitratu* [58, 59]. The increased nutritional benefits from a high degree of provisioning can increase their ability to cope with parasite infestation. On the other hand, increased contact with humans, trash, and defecation increase the chance of transmission of alien endoparasites to wild animals [60–63]. It is evident from our findings that of all the ecological variables and geographical locations, that human-dominated landscapes, like urban areas, are the reservoirs of many species of endoparasites. Further, our interactions with the local people also reveal that monkey relocations usually happen in a low profile, avoiding their documentation in media and official records. Thus the information on the relocation of primates is not publicized and therefore not readily available. However, the available data on relocations indicate that bonnet macaques are often relocated either from temples, villages, crop fields or urban areas to wild habitat, without any screening for diseases or endoparasites. Relocation of such commensal animals infected with multiple endoparasites in high abundance is indeed transferring the alien endoparasites to the wild [64]. Although the multiple endoparasite taxa in the natural host body is a rule of nature [65, 66], their higher density can be lethal for an individual and a population [67–69]. The endoparasites become lethal to the host animal only if favorable conditions are available, as when the immunity level of individuals become very weak due to age, poor food resources or sudden exposure to alien endoparasite taxa [70–73]. The infection by multiple endoparasite taxa in an individual can lead to interspecific competition for space and food that can lead to blood loss, tissue damage, abortion, congenital malfunctions and death of the host animal [68, 74–77].

Contrasting seasonality in endoparasite prevalence has been reported in different primate species, e.g., the high prevalence of endoparasites in five species of lemurs was reported in the dry season [78], whereas a high prevalence of endoparasites in the wet season was reported in *Pan troglodytes* [79] and *Mandrillus sphinx* [49]. The higher moisture in the environment is expected to favor endoparasite diversity thus their prevalence may be expected to be higher in the wet season than in the dry season [79]. However, less resource availability increases the ranging and exploration rate that causes stress which in turn helps the endoparasite to multiply. Thus the prevalence of endoparasites may be favored in the dry season [78], this may be the reason for higher endoparasite infection in the dry season in bonnet macaques.

Among different age-sex individuals of primate society, females are known to have a high infection of endoparasites than males, e.g., *Pongo abelii* [64], *Papio cynocephalus* [80], *Procolobus rufomitratus* and *Cercocebus galeritus* [81]. Interactions of males with the group are usually restricted to the mating and when fighting [82, 83]. Thus the infection rate may be relatively less than females and immatures. However, female bonnet macaque shows relatively high infection of protozoans only in the monsoon season and not in other seasons. Protozoans are waterborne, and they multiply and persist during the rainy season [84]. Thus their infection also may be more prevalent during the monsoon season than in the dry season. It is unclear that the infection of protozoans is higher in females than in other individuals. The higher infection of the endoparasite was reported in immature of *Papio anubis* [85] and *Macaca fuscata* [86]. Similarly, although statistically not significant but immature bonnet macaque had a higher infection of helminths than adults. It is evident that their immune system will be under development and further, since they also spend more time on exploration and play, increases the chance of getting infected.

In spite of guidelines available for relocation of animals, the relocation of a common species like bonnet macaques is often done without following them. This can lead to unexpected impacts on populations of sensitive species in the wild and is a management concern. Since the prevalence of endoparasites persists throughout the year, and that groups exposed to human-dominated landscapes, especially urban and temple groups, translocated animals are likely to carry high endoparasite loads. Proper screening and treatment before relocating to another habitat are required. The entire group has to be captured, the captured animals should be screened for endoparasite and other diseases, they should be treated if they are infected with any endoparasites and, until the animals are free of any infection, they have to be properly maintained by providing food and medical treatment. Once the animals are free of infection, the entire groups should be released at appropriate locations. However, the relocation of any captured groups should be released to a habitat, like protected areas with forest-dwelling animals, and further, we suggest relocating to wild habitat should be avoided or discouraged. We also suggest the strict implementation of guidelines by Woodford [87] for the relocation of all the common species especially the commensal animals including the bonnet macaque.

## Supporting information

S1 Table Test of autocorrelations (Spearman’s rank correlation test) between five independent variables (altitude, group size, vegetation and provisioning) used in GLM to predict the distribution of endoparasites in sampled bonnet macaque groups (N = 20 for all the correlation tests). (DOCX)

S2 Table Data on relocation of primates in India and source of information. (DOCX)

S3 Table Data on endoparasites detected and their number in spatial samples of bonnet macaque. (XLSX)

S4 Table Data on endoparasites detected and their number in temporal samples of bonnet macaque group in Chiksuli, Central Western Ghats. (XLSX)
