# Supplementary material for: Prevalence of gastrointestinal parasites in bonnet macaque and possible consequences of their unmanaged relocations
Source: PLoS One. 2018 Nov 15;13(11):e0207495. doi: 10.1371/journal.pone.0207495 (PMC6237399; doi:10.1371/journal.pone.0207495)
Supplement: S1 Table — (DOCX) [file pone.0207495.s001.docx]

S1 Table. Test of autocorrelations (Spearman’s rank correlation test) between five independent variables (Altitude, Group Size, Vegetation and Provisioning) used in GLM to predict the distribution of endoparasites in sampled bonnet macaque groups (N=20 for all the correlation tests)

|  |  | Group size | Habitat/Vegetation | Group type | Degree of provisioning |
| --- | --- | --- | --- | --- | --- |
| Altitude | r | -0.24 | 0.30 | 0.12 | -0.16 |
|  | p | 0.31 | 0.20 | 0.60 | 0.50 |
| Group size | r |  | -0.10 | 0.03 | 0.40 |
|  | p |  | 0.68 | 0.90 | 0.08 |
| Vegetation | r |  |  | 0.46 | 0.30 |
|  | p |  |  | 0.04^*^ | 0.20 |
| Group type | r |  |  |  | 0.37 |
|  | p |  |  |  | 0.11 |
