# Supplementary material for: Prevalence of gastrointestinal parasites in bonnet macaque and possible consequences of their unmanaged relocations
Source: PLoS One. 2018 Nov 15;13(11):e0207495. doi: 10.1371/journal.pone.0207495 (PMC6237399; doi:10.1371/journal.pone.0207495)
Supplement: S2 Table — (DOCX) [file pone.0207495.s002.docx]

S1 Table. Records of relocation of different primate species in India in the last three decades (between 1989 and 2017)

| **Species** | **Location** | **Reason for translocation** | **Year of Incidence** | **Capturing** | **Technique used to capture** | **Duration of capturing-releasing** | **Initial group size** | **Number of animals trapped** | **Location of release** | **Examined for health, diseases, and parasites** | **Monitored after the release** | **Habitat of the release location** | **Source*** |
| --- | --- | --- | --- | --- | --- | --- | --- | --- | --- | --- | --- | --- | --- |
| BM | Attakatti, Tamil Nadu | Steel-Snatch-Bite | 2000 | Forest Dept. | Cage | 3 days | >50 | ~20 (skewed) | Akkamalai | No | No | Evergreen >1200 m asl | Our Observation |
| BM | Aliyar-Attakatti/Tamil Nadu | Steel-Snatch-Bite | 2000 | Forest Dept. | Cage | No Inf. | >40 | >15 (skewed) | Manomboli/Akkamalai | No | No | Evergreen >1200 m asl | Our Observation |
| BM | Adichunchanagiri /Karnataka | Steel-Snatch-Bite | 2001 | Local people/ trappers | Cage | 1 week | >60 | Released skewed age-sex individuals in different places | Melkote/ Mandya main road | No | No | Habitat/ but PA | Our Observation |
| BM | Chamundi Hill/Karnataka | Steel-Snatch-Bite | 2003 | Forest Dept. | Cage | 3 days | 40-60 | ~20 (skewed) | Balle | No | No | Habitat/ but PA | Our Observation |
| BM | University of Mysore Campus/Karnataka | Perceived nuisance | 2004 | Forest Dept. | Cage | 5 days | ~30 | 8-9 (skewed) | HD Kote road | No | No | Roadside/close by forest | Our Observation |
| BM | Outskirts of Kollegala town/Karnataka | Perceived nuisance | 2004 | Local people/ trappers | Cage | No Inf. | No Inf. | ~30 animals | Foot hills of MM hills | No | No | Roadside/close by forest | Our Observation |
| BM | Agriculture fields of Kollegala/Karnataka | Crop damage | 2004 | Local people/ trappers | Cage | No Inf. | No Inf. | ~30 animals, transported by gunny bag, died due to suffocation | Foot hills of MM hills | No | No | Habitat/ but PA | Our Observation |
| BM | Agriculture fields near Sirsi/Karnataka | Crop damage | 2002 | Local people/ trappers | Cage | No Inf. | No Inf. | ~30 | Devimane Ghat | No | No | EG | Our Observation |
| BM | Ramnagara temple/Karnataka | Crop damage | 2004 | Local people/ trappers | Thread Snares | No Inf. | ~50 | ~15 | 20 km from Ramnagara/ main road | No | No | Roadside/close by forest | Our Observation |
| BM | Agriculture fields and villages -around Karkala/Karnataka | Crop damage | 2011 | Local people/ trappers | Cage | No Inf. | No Inf. | ~12 (animals, transported by gunny bag, died due to suffocation) | Kudremukh foot hills | No | No | EG | Our Observation |
| RM | Aligarh Muslim University/Uttar Pradesh | Steel-Snatch-Bite | 1995 | Local people/ trappers | Cage | No Inf. | No Inf. | 7 | Tughlaqabad Fort, South Delhi | No | No | No Inf. | Imam and Yahya (2002) |
| RM | Aligarh Muslim University /Uttar Pradesh | Steel-Snatch-Bite | 2001 | Local people/ trappers | Cage | No Inf. | 27 | 22(skewed ) | Semi natural forest of Jawan Aligarh | No | No | Roadside/close by forest | Imam and Yahya (2002) |
| HG | Dello, North east India | To save from hunting in the fragment | 2011 | Forest Dept. | Cage | 1 day | 2 | 2 | Mehao Wildlife Sanctuary | No | No | EG | WTI report January 2011 |
| BM | 15 Senani puram, Tiruvananthapuram /Kerala | Crop damage | 2014 | Local people/ trappers | Poisoned | 1 day | No Inf. | 15 | Poisoned to death | No | Died | Plantation | Times of India January 13 2014 |
| RM | Vrindavan/ Uttar Pradesh | Steel-Snatch-Bite | 2002 | Forest Dept. | Cage | No Inf. | 1,338 | 600 (skewed) | 5-45 km forest | No | Yes | Scrub forest | Imam et al. (2002) |
| RM | Delhi | Perceived nuisance | 2004 | Forest Dept. | Cage | No Inf. | 30,000 | 500 (skewed) | Pilibhut and Kuno National Park. | No | No | Habitat/ but PA | Janaki Menon, The Hindu , Oct 2 2005 |
| RM | Sariska Tiger Reserve/Rajasthan | spreading the parasitic infections to the wild animals | 2000 | Forest Dept. | Cage | No Inf. | No Inf. | 300 (skewed) | Dholpur and Bharatpur forest area | No | No | Scrub forest | Protected Area Update ( August 2000) |
| BM | Aliyar /Tamil Nadu | Steel-Snatch-Bite | 2005 | Forest Dept. | Cage | No Inf. | No Inf. | 60 (skewed) | Nearby forest | No | No | Evergreen >1200 m asl | Kumar (2006) |
| BM | Attakatti /Tamil Nadu | Steel-Snatch-Bite | 2005 | Forest Dept. | Cage | No Inf. | No Inf. | 300 (skewed) | Nearby forest | No | No | Evergreen >1200 m asl | Kumar (2006) |
| RM | Shimla Surrounding/Himachal Pradesh | Steel-Snatch-Bite | 2004-2006 | Forest Dept. | Cage | No Inf. | No Inf. | 3400 (skewed) | Nearby forest | No | No | Conifer forest | Paul (2006) |
| RM | Himachal Pradesh | Steel-Snatch-Bite | 2006-2017 | Local people/ trappers | Cage | No Inf. | No Inf. | 125266 (skewed) | At different places | No | No | Conifer forest | Himachal Pradesh Forest Dept. (2017) |
| RM | Tuglaqabad air force station/ New Delhi | Steel-Snatch-Bite | 1989 | Forest Dept. | Cage | No Inf. | No Inf. | 21 | Rural area of Meethapura ~ 10 km | No | No | Scrub forest | Malik and Johnson (1989) |
| RM | Chatari-do-raha/ Uttar Pradesh | Steel-Snatch-Bite | 1994 | Forest Dept. | Cage | No Inf. | No Inf. | 20 | Canal bank forest- Sumera in Aligarh dt | No | No | Scrub forest | Southwick and Siddiqi (1994) |
| RM | National Zoological Park/ New Delhi | Steel-Snatch-Bite | 1995 | Forest Dept. | Cage | No Inf. | Five groups of 40 animals | 40 | Tughlaqabad Fort, South Delhi | No | No | Scrub forest | Imam and Malik (1997) |
| RM | Air force station, Gurgaon/Haryana | Steel-Snatch-Bite | 1998 | Local people/ trappers | Cage | 1 day | 28 | 22 | Forest of Firozpur- Jhirka | No | Yes | Scrub forest | Imam et al. (2001) |

Sources*

Imam E, Yahya HAS, Malik I. A successful mass translocation of commensal Rhesus monkeys *Macaca mulatta* in Vrindaban, India. Oryx. 2002; 36:87-93.

Imam E, Malik I. Translocations of monkeys from National Zoological Park, New Delhi to Tughlaqabad Fort, South Delhi. New Delhi: Report submitted by Vatavaran to the National Zoological Park; 1997.

Southwick CH, Siddiqi MF. Population status of non-human primates in Asia, with emphasis on rhesus macaques in India. Am J Primatol. 1994; 34:51–59.

Imam E, Yahya HSA. Management of monkey problems in Aligarh Muslim University campus, Uttar Pradesh. Zoos' Pint. 2002; 171:685-687.

Imam E, Malik I, Yahya HSA. Translocation of Rhesus monkey from Airforce Station, Gurgaon Haryana to natural forests of Firozpur-Jhirkha Haryana, India. J Bombay Nat Hist Soc. 2001;983: 355-359.

Malik I. Population growth and stabilizing age structure of the Tughlaqabad rhesus macaque. Primates. 1989; 30: 117-120.

Malik I, Johnson RL. Trapping and conservation: development of a translocation in lndia. ln: A. Ehara A, Kimura T, lwamoto M. editors. Primatology Today. Amsterdam: Elsevier; 1991, pp. 63-64.

Wildlife Trust of India. 2011; <http://www.wti.org.in/news/eighth-family-of-endangered-eastern-hoolock-gibbons-successfully-translocated-to-mehao-wildlife-sanctuary/>.

Protected Area Update, August 2000; <http://www.kalpavriksh.org/images/PAUpdate/ProtectedAreaUpdate26%20_Aug2000.pdf>.

Kumar MA. Translocation: Experiences from the Anamalai Hills. CBSG/RSG South Asia 3^rd^ Annual Joint Meeting, Zoos' Print, 2006; 21(3): RNI 7:6

Paul SK. (2006) Monkey Menace in Himachal Pradesh. CBSG/RSG South Asia 3^rd^ Annual Joint Meeting, Zoos’ Print, 2006; 21 (3): RNI 7:6

Himachal Pradesh Forest Dept. Monkeys Sterilization Programme. <http://hpforest.nic.in/pages/display/NjU0c2RhiHFzZGZhNQ==-monkey-sterilization-programme>. 2017.
